# Integrating terrestrial scavenging ecology into contemporary wildlife conservation and management

**DOI:** 10.1002/ece3.9122

**Published:** 2022-07-17

**Authors:** Jessica R. Patterson, Travis L. DeVault, James C. Beasley

**Affiliations:** ^1^ Savannah River Ecology Lab, Warnell School of Forestry and Natural Resources University of Georgia Aiken South Carolina USA

**Keywords:** ecosystem health, food web dynamics, scavenging ecology, wildlife conservation, wildlife diseases, wildlife management

## Abstract

Scavenging plays a vital role in maintaining ecosystem health and contributing to ecological functions; however, research in this sub‐discipline of ecology is underutilized in developing and implementing wildlife conservation and management strategies. We provide an examination of the literature and recommend priorities for research where improved understanding of scavenging dynamics can facilitate the development and refinement of applied wildlife conservation and management strategies. Due to the application of scavenging research broadly within ecology, scavenging studies should be implemented for informing management decisions. In particular, a more direct link should be established between scavenging dynamics and applied management programs related to informing pharmaceutical delivery and population control through bait uptake for scavenging species, prevention of unintentional poisoning of nontarget scavenging species, the epidemiological role that scavenging species play in disease dynamics, estimating wildlife mortalities, nutrient transfer facilitated by scavenging activity, and conservation of imperiled facultative scavenging species. This commentary is intended to provide information on the paucity of data in scavenging research and present recommendations for further studies that can inform decisions in wildlife conservation and management. Additionally, we provide a framework for decision‐making when determining how to apply scavenging ecology research for management practices and policies. Due to the implications that scavenging species have on ecosystem health, and their overall global decline as a result of anthropic activities, it is imperative to advance studies in the field of scavenging ecology that can inform applied conservation and management programs.

## Graphical abstract

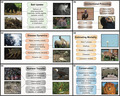

## 1 INTRODUCTION

Scavenging is an important ecological function that plays a vital role in maintaining ecosystem health by stabilizing food webs (DeVault et al., 2003; Wilson & Wolkovich, 2011), reducing disease transmission by decreasing the time host reservoirs are in contact (Ogada, Torchin, et al., 2012), and increasing nutrient transfer between environments (Cederholm et al., 1999) across the globe. Research in this growing sub‐discipline of ecology continues to develop our understanding of the role of carrion and scavenging in ecological processes. One topic of concern is the influence of anthropic activities on scavenger species, and its detrimental effects to ecosystems by altering competitive interactions between microbes, invertebrates, and vertebrates and reducing ecosystem services and functions (Beasley et al., 2015; Sebastián‐González et al., 2019). Similarly, wildlife management and conservation practices can have both direct and indirect impacts to scavengers, which can affect trophic interactions within food webs (DeVault et al., 2003; Wilson & Wolkovich, 2011). In addition to the impacts of anthropic activities, it is important to consider the role of scavenging in other areas that may appear unrelated, such as documenting wildlife mortalities and estimating mortality correction factors for detectability and carcass removal (Smallwood et al., 2010; Teixeira et al., 2013), and understanding how aquatic nutrients can be moved across terrestrial landscapes by scavengers (Cederholm et al., 1999).

**FIGURE 1:**
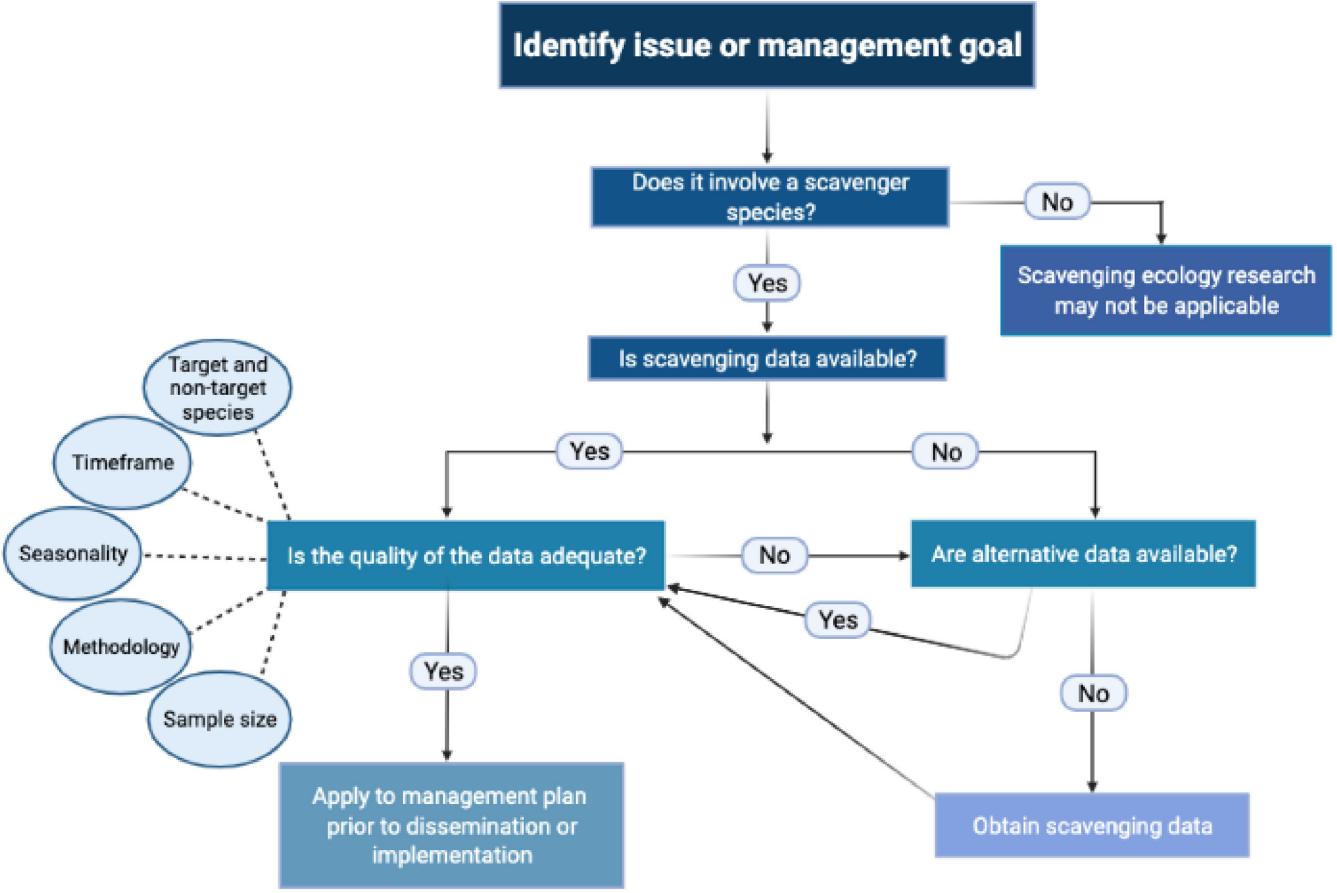
A decision‐making framework for determining when and how to integrate scavenging research into management and conservation planning

Although research in this area is growing, there is an apparent disconnect between scavenging ecology and its use in the application of wildlife conservation and management practices (Mateo‐Tomás et al., 2019; Newsome et al., 2021). Further, it has become apparent that immediate action should be taken to conserve scavenger species and develop policies for managing carrion, particularly from farming, hunting, and fishing discards (Mateo‐Tomas & Olea, 2018). This review aims to compile and summarize the specific areas where scavenging ecology can be further integrated into management and conservation plans and applications. For example, although there has been a surge of research investigating mortalities of birds and mammals at wind farms in response to the acceleration of wind energy production (Johnson et al., 2002), there has been little effort to elucidate scavenging dynamics associated with animal mortalities at these facilities, despite the application of such information to management decision making (DeVault et al., 2017). Similarly, as human populations increase, road use and wildlife vehicular mortality often increases as well. Hill et al. (2020) noted that vehicle mortality of North American mammals has increased 4‐fold in the last five decades (1965–2017), highlighting the need for scavenging research during roadway and bridge development and planning. Given the declining populations of many scavenger species globally, coupled with the underrepresentation of scavenging ecology in the literature (Olea et al., 2019), there is a need for more explicit integration of scavenging studies into the developmental stages of wildlife conservation and management strategies. Our objective is to review the scientific literature to identify areas within the broad topics of poisoning, bait uptake, conservation, wildlife mortalities, wildlife diseases, and nutrient transfer where scavenging ecology research should be focused for implementation of contemporary wildlife conservation and management practices. In addition, we provide a framework for determining if and how scavenging research can be applied to management practices and policies (Figure 1). We focus our review on the delivery of pharmaceuticals through baiting operations, poison bait uptake by nontarget scavengers during nuisance wildlife control operations, the epidemiological role that scavengers play in wildlife disease dynamics, documenting wildlife mortalities to ensure an accurate count of carcasses, the transfer of nutrients through scavenging, and conservation of imperiled scavengers.

## 2 METHODS

We performed a systematic literature search to obtain English language peer‐reviewed scientific articles for publication dates up to and including April 2022. The search was conducted in Google Scholar and Web of Science by combining the following keywords: “scavenging ecology*” and “management*”, “nontarget species*” and “poison*” and “bait uptake*”, and “scavenging species*” with “bait uptake*”, “conservation*”, “carcass counts*”, “wildlife mortalities*”, “wildlife diseases*”, and “nutrient transfer*”. Following the recommendations of Haddaway et al. (2015), we focused within the first 300 Google Scholar results to include gray literature in this review. When we found a study related to these topics, we included related manuscripts from the literature cited section and literature that referenced that publication. This search yielded 107 papers that we reviewed for this study. Our primary goal was to find articles on scavenging ecology studies and knowledge gaps in the literature pertaining to scavenging species or ecosystem processes linked to scavenging that could potentially be applied to future studies to inform conservation and management decisions. Therefore, this is a review that compiles relevant scavenging ecology studies with a guide for application of scavenging research in conservation and management decisions.

## 3 BAIT UPTAKE FOR SCAVENGERS

The practice of bait uptake for pest eradication, invasive species population control, and delivery of pharmaceuticals is used globally for a broad range of taxa such as foxes (Trewhella et al., 1991), cats (Short et al., 1997), rodents (Brunton et al., 1993), wild pigs (*Sus scrofa*; Beasley et al., 2021; Cowled et al., 2007), and many other species. In particular, oral vaccination programs have been established to manage rabies in numerous carnivore species such as red foxes (*Vulpes vulpes*) in Europe (Brochier et al., 1990) and raccoons (*Procyon lotor*) in the US (Slate et al., 2009). Similarly, baiting programs have been used for the control of parasites, such as *Baylisascaris procyonis* in Allegheny woodrats (*Neotoma magister*; Page et al., 2011), and to protect wild Tasmanian devils (*Sarcophilus harrisii*) from devil facial tumors (Dempsey et al., 2022). Many of these programs incorporate species‐specific flavor preferences into the formulation of bait matrices. However, baiting efforts for facultative scavengers could benefit from a broader integration of assessments routinely quantified through studies of scavenging dynamics, such as behavioral interactions with baits, time to detection, interspecific interactions and competition for baits, and abiotic and biotic factors affecting bait acceptance and detection.

**FIGURE 2:**
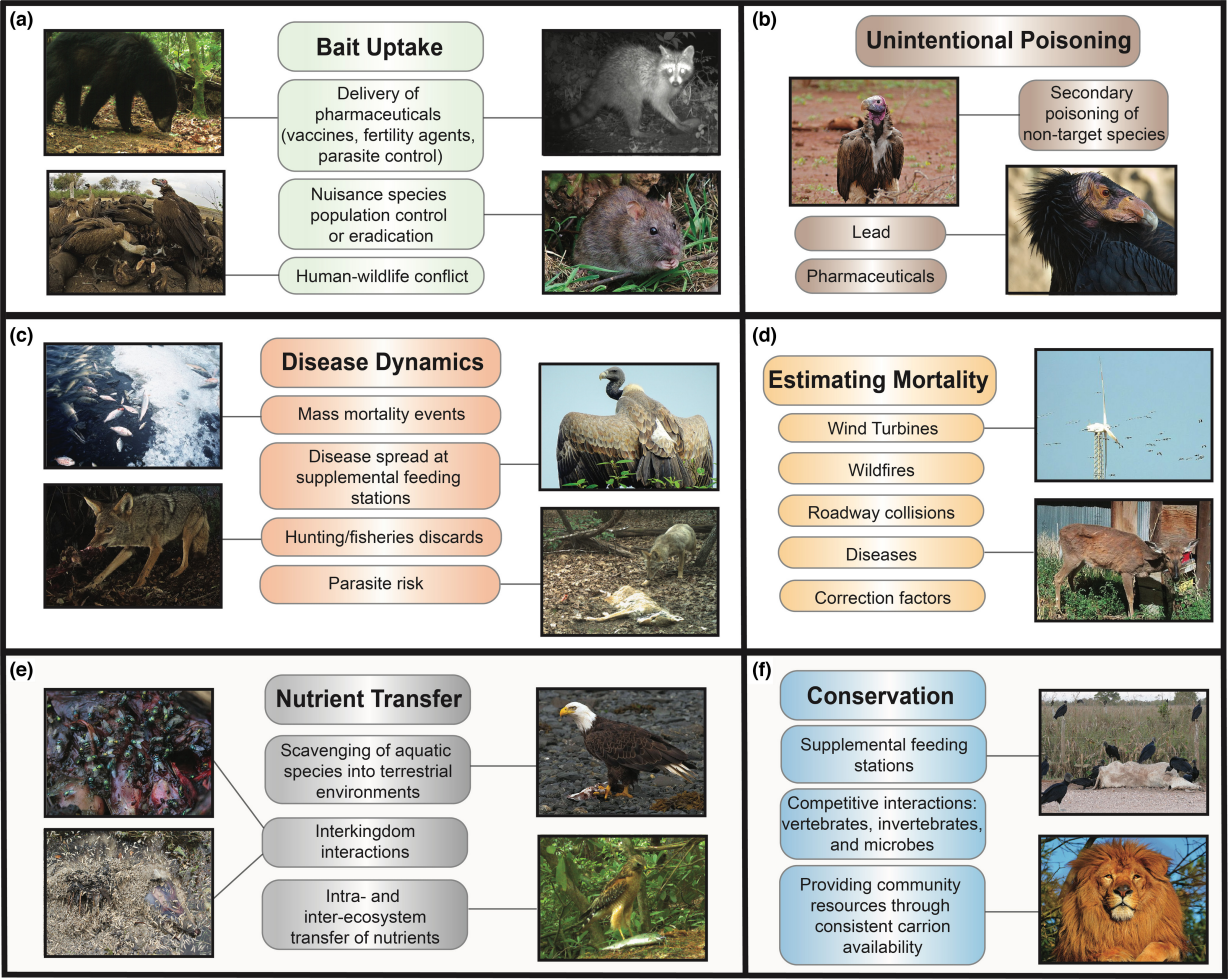
Recommendations of scavenging ecology studies for wildlife conservation and management practices. Photo credits: (a) black bear and raccoon (Jessy Patterson), vultures on elephant carcass (C fallows, AJ Gallaghers, N Hammerschlag, CC License), Norway rat (reg McKenna, CC License). (b) Lappet‐faced vulture (Bernard Dupont, CC License), California condor (chuck Szmurlo, CC License). (c) Fish die‐off (USFWS, CC License), Indian vulture (Shantanu Kuveskar, CC License), coyote feeding on deer discards (NPS, CC License), coyote investigating coyote carcass (Miranda Butler‐Valverde). (d) Wind turbines (Raju Kasambe, CC License), CWD deer (Terry Kreeger, CC License). (e) Blow flies on porcupine carcass (Paul venter, CC license), maggots on opossum carcass (Tim Vickers, CC license), bald eagle (Yathin S. Krishnappa, CC license), red‐shouldered hawk (Jessy Patterson). (f) Vultures on cow carcass (Bernard Dupont, CC license), lion (Clement Cardot, CC license)

Another area primed for bait uptake research focuses on invasive vertebrate scavengers, which have been introduced to all parts of the world. Although some non‐native species provide resources and economic gains for humans, others cause serious detrimental effects such as the spread or introduction of diseases, environmental degradation, and competition with or predation of native species. In the US alone, hundreds of vertebrate species have been introduced and have established breeding populations (Pitt et al., 2018), causing environmental damages and losses up to $120 billion per year (Pimentel et al., 2005). It is estimated that invasive species are involved in 86% of extinctions of island species and are endangering hundreds of extant vertebrate species (Spatz et al., 2017). Many of these invasive mammals, including rats (*Rattus sp*.), pigs, cats (*Felis catus*), dogs (*Canis familiaris*), and mice (*Mus sp*.), are facultative scavengers that may be eradicated by poison bait uptake. For example, feral cats are eliminating native species on Little Cayman Island in the Caribbean. The Department of Environment and Department of Agriculture intend to trap and humanely euthanize all feral cats (Department of Environment, 2021); however, if the feral cat population requires additional measures for eradication, a scavenging study to assess bait type, bait flavor, the impact on non‐target species, and other criteria as outlined above is recommended prior to implementation of a plan. Additionally, along with red foxes, it is estimated that feral cats kill over 2 billion animals per year in Australia (Stobo‐Wilson et al., 2022). Invasive vertebrates tend to be highly efficient facultative scavengers (Abernethy et al., 2016), so once a species is established, management is often focused around eradication and control (Genovesi, 2005), and scavenging studies can be used to guide decision‐making (Figure 2a). We suggest that studies exploring biotic and abiotic factors influencing carcass consumption across scavenger species (DeVault et al., 2017; Stiegler et al., 2020) become more routinely integrated into the management planning process to help managers pinpoint the most effective delivery of bait or pharmaceuticals for target species.

## 4 UNINTENTIONAL POISONING OF SCAVENGERS

### 4.1 Nontarget species

Poison baits are used worldwide for nuisance wildlife control, yet, can be consumed by nontarget species, primarily scavengers that are susceptible to secondary poisoning by feeding on poisoned carcasses of the target or other nontarget species (Figure 2b). There is a common misconception that species succumbing to toxicants may perish in areas inaccessible to scavengers and be decomposed by invertebrates and microbes (Howald et al., 1999); however, there is growing awareness that carcasses of poisoned animals are often readily accessible and consumed by vertebrate scavengers (Ogada et al., 2016; Smith et al., 2016) and even carcasses suspended in vegetation or underground are often scavenged by vertebrates (DeVault & Krochmal, 2002).

Anticoagulant rodenticides (ARs) are used globally in poison baits for rodent population control, and although some risk mitigation measures have been instituted, such as safe disposal of poisoned rodents and tamper‐resistant bait boxes (Buckle & Prescott, 2018), the efficacy of these measures and sublethal and nontarget impacts of rodent control are often unknown. Koivisto et al. (2018) investigated the effects of ARs on scavenger and predator species in Finland, and discovered AR residues in 82% of the liver samples taken. Similarly, Montaz et al. (2014) compared seasonality and richness of species scavenging rodents exposed to ARs in France. They concluded multiple scavenging species were present in their study and vulnerable to AR exposure; but raptors, particularly the red kite (*Milvus milvus*) and common buzzard (*Buteo buteo*), both protected species in Europe, may be declining in numbers due to rodenticides after consuming poisoned rodent carrion. These and other studies evaluating effects of poison bait uptake on nontarget species are often undertaken after eradication plans have been implemented (Howald et al., 1999; Hughes et al., 2013). However, we advocate the incorporation of scavenging studies into the developmental process of management practices for nuisance species, prior to field implementation of management regimes using toxicants, to proactively mitigate effects on nontarget species. For example, a feral cat (*Felis catus*) eradication study was conducted in Australia using non‐toxic baits to assess uptake by nontarget species before deploying toxic baits (Hohnen et al., 2020). They concluded that 99% of identifiable bait takes were consumed by nontarget species, including several endangered species, indicating an alternative bait should be considered for feral cat eradication. Furthermore, many poisons can bioaccumulate (Geduhn et al., 2015), highlighting the complexities of how bait uptake can affect food web dynamics, apex predators, and ecosystem functions. Such proactive assessments of nontarget impacts of poisoning campaigns are infrequent; however, such assessments are vital in the developmental stages of contemporary management plans for nuisance species. For example, eradication efforts for mice in the South Farrallon Islands, USA are currently in the planning process (U.S. Fish and Wildlife Service, 2021). The goal is to restore local endemic populations of species that are decimated by mice and rat populations (e.g., camel crickets, arboreal salamanders, plants) and petrels that are preyed upon by owls that are attracted to the island by mice and rats. Prior to dissemination of AR‐laced grain pellets throughout the island for rodents to feed on, scavenging studies to determine the use of dead rodents by scavengers could be useful in preventing non‐target species consumption and potential bioaccumulation of local species.

As the only terrestrial obligate vertebrate scavengers, vultures are especially vulnerable to the effects of unintentional poisoning, and many vulture species are in decline and endangered. Although the causes of vulture declines are complex and multifaceted, intentional and unintentional poisoning remains a top threat to the recovery of populations. For example, poisoning of Asian (*Gyps*) vultures through ingestion of nonsteroidal anti‐inflammatory drugs administered to livestock, particularly diclofenac‐sodium, reduced the vulture numbers by over 95% (Green et al., 2004). In 2006, the India government enforced a ban on production, importation, and sale of diclofenac products that slowed vulture population declines. However, diclofenac is still used in other countries and without adequate regulation there could be detrimental effects to other vulture populations (Margalida et al., 2014). In Africa, human‐wildlife conflict between farmers and megafauna led to the poisoning of more than 400 vultures; unintentionally through the consumption of poisoned baits or poisoned carcasses, and intentionally by poachers to prevent detection (Ogada et al., 2016; Safford et al., 2019). These circumstances have elucidated the detrimental effects that declining vulture populations can have on ecosystem services, economic activity, and human health (Markandya et al., 2008; Morales‐Reyes et al., 2017).

### 4.2 Lead poisoning

Unintentional lead poisoning through ingestion of spent shot and bullets has similarly been identified as a threat to many raptors, including the critically endangered California Condor (*Gymnogyps califonianus*; Figure 2b), declining Andean Condor (*Vultur gryphus*), and Old World vultures (Griffon vulture; *Gyps fulvus*), as well as mammals (Mctee et al., 2019). Lead exposure can result in reduced fecundity, increased bone fragility, and higher susceptibility to infection (Garvin et al., 2020). To date, 33 countries have implemented restrictions on the use of lead ammunition to mitigate this problem (Garvin et al., 2020), and efforts should be made to determine the efficacy of these legislative actions. Recently, Ellis and Miller (2022) published results determining the efficacy of the lead ammunition ban in Illinois, USA, indicating a reduction in crippling rates for both ducks and geese after implementation of the ban. These results counter the expectations of many hunters and show a positive and unexpected outcome for lead ammunition bans. In addition, Green et al. (2022) found lower levels of lead in raptor liver tissues in Denmark compared with data from countries without a ban, including pre‐ban Denmark. We recommend similar studies be applied across the globe and considered when discussing implementation of lead ammunition restrictions.

Due to the worldwide decline of scavenger species and the imperative roles they play in ecosystems (DeVault et al., 2016), we must focus our attention on understanding how various methods for unintentional poisoning can alter food web dynamics and ecosystem function. Additional studies, such as those exploring optimal bait types and bait distribution strategies that minimize impacts to nontarget species, should be prioritized in any control strategies implementing toxicants (Snow et al., 2018). We also recommend further exploration into alternative strategies of wildlife control other than employing toxicants, such as lights, noises, and electric fences (Lozano et al., 2019), as well as further research into contaminant and toxicant biomagnification in scavenging species, and sub‐lethal effects of contaminant/toxicant exposure to scavengers and other wildlife.

## 5 DISEASE IMPLICATIONS

Another important area of research primed for growth is the epidemiological role that scavenging species play in disease dynamics, for both wildlife and humans. There are still many knowledge gaps relative to the effects different species have on disease dynamics and the underlying conditions and circumstances in which scavenging enhances or suppresses disease spread (Figure 2c).

Carrion is available and sometimes abundant throughout various regions across the globe, accumulating large amounts of animal biomass through natural mortality, predation, mass die‐offs (e.g., from natural disasters, algal blooms, diseases, spawning salmon), and human provisions (e.g., culling, hunting/fisheries discards; Moleón et al., 2019). Surpluses of carcasses on a landscape, sometimes abrupt and massive, can increase potential for pathogen spread, and are increasing in frequency with global change (Thomas et al., 2004). Regardless of how carrion is generated, both vertebrate and invertebrate scavenging species often reduce the time in which a carcass is decomposing on the landscape, reducing the time available for diseases to spread (Hill et al., 2018; Mackey & Kribs, 2021). Invertebrate scavengers are particularly productive in carrion mass loss, accounting for the removal of up to 90% of tissues from vertebrate carcasses within several days (Payne, 1965). Consequently, there can be a cascading effect resulting in rampant disease spread when scavenger species are removed from the landscape. For example, the Asian vulture crisis in India resulted in a >95% decline in vulture numbers. As a result, feral dogs became the primary consumer of carcasses and their population numbers increased. As feral dogs are a main reservoir for rabies, this may have resulted in a higher rate of virus transmission and increased human risk for infection (Markandya et al., 2008; Ogada et al., 2016).

There are still many unanswered questions that should be considered for future scavenging studies that can inform conservation and management decisions, especially regarding mass die‐offs and pulses in carrion resources. Very few studies have focused on mass mortality events (MMEs) due to their unpredictable nature, specifically how they affect scavenging communities, disease spread, and ecosystem health (Tomberlin et al., 2017). Consequently, we encourage future studies explore the influence of scavenging species on disease dynamics at MMEs and changes in vertebrate and invertebrate behavior as a result of MMEs (Frank et al., 2020), particularly given the anticipated increased frequency of these events due to climate change and other anthropogenic factors. Additionally, due to their unpredictability, we encourage consideration of integrating simulated MMEs into future studies, as that allows replication and gathering of data on initial conditions of the environment prior to the MME. Further, simulations provide an opportunity to control the environment (i.e., fencing the area to prevent vertebrate scavengers and assess only invertebrate activity) to assess ecosystem processes without confounding factors (Lashley et al., 2018).

Although scavengers provide ecosystem services by removing decomposing carcasses (Grilli et al., 2019; Markandya et al., 2008), in some circumstances, scavengers also might act as pathogen vectors by transporting infectious materials to other areas. For example, though vultures are thought to be particularly well‐suited to inhibit disease spread when consuming carrion by utilizing highly acidic stomach secretions that destroy nearly all microbes (Houston & Cooper, 1975) and might greatly reduce the chance of infection from a decomposing carcass (Ogada, Keesing, & Virani, 2012), some microbes can survive the vulture digestive tract and be regurgitated or passed through feces (Houston & Cooper, 1975). Additionally, more recent studies show the pH of New World vulture stomach secretions are no more acidic than non‐scavenging avian species and domestic fowl (Graves, 2017). It is also speculated, but has not been previously investigated, that vultures and other migratory birds can carry pathogenic organisms from carrion sites on their feet, potentially facilitating disease spread (Ogada, Keesing, & Virani, 2012). Likewise, increased pathogen transmission rates between hosts at supplemental feeding stations (SFS) have been reported (Murray et al., 2016), and vultures are likely infected by zoonotic *Salmonella* strains from carcasses provided at SFS (Marin et al., 2018). Finally, VerCauteren et al. (2012) concluded that American crows (*Corvus brachyrhynchos*), a common facultative scavenger, are able to pass infectious prions in their feces after consuming prion‐positive tissues, such as those from transmissible spongiform encephalopathy (TSE) diseases including chronic wasting disease, scrapie, and bovine spongiform encephalopathy. Alternatively, it was previously speculated that scavenging species played a critical role in the spread of anthrax by scavenging infected carcasses, but now it is understood they do not increase transmission (Bellan et al., 2013), adding to the complexities of our understanding of scavenging and disease dynamics.

As outlined above, available evidence suggests scavengers act to suppress disease spread overall, but it is unclear whether scavenging species might contribute to the spread of diseases in some circumstances, and thus this remains an area where additional research is critically needed. Avian scavengers, in particular, could facilitate the spread of disease between SFS, and given that there are successful “vulture restaurants” in countries such as Nepal and India, we recommend further studies be conducted in controlled environments like these to provide data that can inform decision‐making in future SFS management policies and practices. Further, Theimer et al. (2017) found the rabies virus was transmitted to scavenging mesocarnivores after ingesting infected dead bats. Striped skunks (*Mephitis mephitis*) were the primary scavenger consuming bats, but raccoons, gray foxes (*Urocyon cinereoargenteus*), and domestic cats also consumed bat carcasses, acting as potential vectors for the rabies virus. Although recent studies have elucidated the complexities associated with the landscapes of fear and disgust and scavenging of conspecifics, there are still areas to investigate such as animal responses to signals associated with parasite risk (Gonzálvez et al., 2021), and parasite risk in relation to carcass size, ecosystem type, and season (Moleón & Sánchez‐Zapata, 2021). In addition to vertebrate scavengers, we advocate for further studies on invertebrates that visit carrion, such as carrion flies (Hall et al., 2019), ants, and mosquitoes, for a holistic contribution to understanding disease spread in relation to scavenging.

## 6 DOCUMENTING MORTALITIES

As outlined above, wildlife mortalities occur for many different reasons and result in carcasses distributed across broad landscapes. To understand population declines and anthropogenic causes of mortality, it is imperative to accurately quantify wildlife carcasses. Previous studies have quantified carcass counts for various species or taxonomic groups due to mortality from many sources including poisoning (Vyas, 1999), roadway collisions (Langen et al., 2007), collisions with infrastructure (Loss et al., 2015), and wildfires (Silveira et al., 1999). Estimating carcass numbers can be complex due to difficulty in detecting carcasses and because carcass removal is likely to occur by vertebrate scavengers or decomposers (Smallwood et al., 2010; Teixeira et al., 2013) before counts can be made. Estimating correction factors is also necessary for accurately documenting wildlife mortalities. However, aside from avian mortalities, few studies estimate correction factors to account for scavenger removal. This is certainly an area primed for further investigation, as scavenging research can play an important role in better understanding mortality rates and estimating correction factors. With increased recognition that scavengers skew carcass quantification and the known application of such information to structured decision‐making for wildlife conservation and management (DeVault et al., 2017), we recommend further studies focus on how specific carcass types are used and how carcass size and habitat can influence scavenging rates to fine‐tune our understanding of the complexities of carrion‐scavenger relationships (Figure 2d). For example, unprecedented wildfires occurred over the last several years across Australia, Russia, the western United States, Brazil, and many more countries across the globe. Carcass counts to quantify the number of species and individuals that succumbed to the fire may be conducted, but an estimation factor to account for removal of carcasses by scavengers would result in a more reliable estimation. Additionally, during the environmental review process for infrastructure project planning for roadway construction, wildlife fencing, crossing structures, and detection systems are often considered to mitigate impacts on wildlife. During assessment of the efficacy of these mitigation plans, particularly those involving wildlife vehicle collisions, scavenging of carcasses and producing estimation factors to account for skewed counts should be considered.

Accurately quantifying carrion biomass is imperative for managing carrion resources, which play a critical role in ecosystem health and function through nutrient cycling and providing food resources for scavenging species. However, quantitative data on carrion biomass are largely lacking, and thus the direct roles that carrion plays in ecosystem health are not fully understood (Barton et al., 2019). In addition to MMEs, there are many incidences resulting in the distribution (or lack thereof) of carrion biomass. For example, recent studies have highlighted the decline in carrion biomass availability (both from livestock and big game) due to sanitary restrictions and regulations imposed on carrion removal (Margalida & Moleon, 2014; Morales‐Reyes et al., 2015). The removal of carcasses reduces a food source for scavenger species and could have detrimental effects on the ecosystem. Additionally, when estimating carrion biomass, it is important to make a distinction between carrion production, or a function of mortality and carrion availability, which depends on carrion production and other factors (i.e., habitat, season, etc.). Very few studies quantify both carrion production and availability in space and time, and should be considered for future research (Moleón et al., 2020). Although little is known about estimating carrion biomass, recent studies have provided a framework that can be implemented for management purposes (Barton et al., 2019; Moleón et al., 2019; Morant et al., 2022); however, additional studies are needed to apply this framework across a broad range of ecosystems. In particular, we recommend the application of this framework to ecosystems or areas with imperiled and/or protected scavenging species, areas with growing populations of ungulates that are recolonizing abandoned rural areas (Morant et al., 2022), and studies focusing on the contribution of invertebrate carcasses to total carrion biomass.

## 7 NUTRIENT TRANSFER

There is a growing understanding of the importance that scavenging plays in connecting food webs (Beasley et al., 2019), and facultative scavenging can result in an estimated 16‐fold increase in food web linkages (Wilson & Wolkovich, 2011). As more linkages are formed, food webs become more connected and stable (McCann, 2000). Although many scavenging links are poorly understood, linkages involving the transfer of nutrients between ecosystems are particularly unclear (Ballinger & Lake, 2006; Cederholm et al., 1999). Scavengers undoubtedly play a role in moving resources and nutrients between adjacent ecosystems, but the degree to which this occurs and the resulting effects on ecosystem health should be investigated further (Schlichting et al., 2019). Also, few data are available discussing the importance of aquatic carrion for survival and reproduction of terrestrial scavengers, and more detailed studies are needed to examine these linkages (Rose & Polis, 1998). This is an area of research primed for growth that has extensive implications for species and ecosystem conservation, and we recommend studies exploring inter‐ and intra‐ecosystem linkages via scavenging and the fate and scavenging rate of aquatic carcasses by terrestrial vertebrates, (Figure 2e).

Food web linkages and interactions between vertebrate scavengers, invertebrate scavengers, and decomposers in terrestrial environments have been addressed in the literature (DeVault et al., 2003, 2004; Tomberlin et al., 2017); however, many questions remain. Understanding how changes in species interactions and carrion food webs could modify ecosystem functions is extremely important, yet, very few studies have compared communities across taxonomic groups (Barton & Bump, 2019). Although it is understood that large apex predators can affect invertebrate assemblages, little is known about the importance of predator‐produced carrion to invertebrates and decomposers (Barry et al., 2019). Another area requiring attention is MMEs and the affects they have on decomposer communities, how massive amounts of carrion in a single landscape modify nutrient cycling, and how plant communities are impacted (Tomberlin et al., 2017). Finally, more studies should focus on interaction pathways between vultures, carrion, large carnivores, and their prey, with emphasis on food‐web dynamics to understand ecosystem stability as vertebrate scavenging populations decline (Moleón et al., 2014). We recommend the incorporation of these topics into future studies to fully elucidate nutrient transfer between scavenger assemblages and decomposers.

Previous studies have focused on investigations of terrestrial inputs into aquatic ecosystems; yet, the flow of aquatic‐derived nutrients into terrestrial ecosystems also can have a significant impact on food web dynamics by altering productivity and predator–prey interactions (Ballinger & Lake, 2006). Salmon carcasses provide marine‐derived nutrients to stream habitats, increasing nutrient contributions to freshwater environments (Cederholm et al., 1999), whereas terrestrial environments can also receive an influx of these nutrients. Reproductive cycles and seasonal distributions of some vertebrate scavengers, such as the bald eagle (*Haliaeetus leucocephalus*; Hunt et al., 1992), mink (*Mustela vison*; Ben‐David, 1997), and grizzly bear (*Ursus arctos horribilis*; Hilderbrand et al., 1996) correspond with spawning of salmon, and salmon‐derived nutrients can have a fertilizing effect on riparian plants through carcass decomposition or deposition of fecal matter (Cederholm et al., 1999). This is still a poorly understood area that requires more focus and could inform management decisions for salmonids within the National Oceanic and Atmospheric Administration (NOAA) Fisheries management plans and the Pacific Fishery Management Council, which subsequently could affect scavenger species and ecosystem health. Furthermore, if ecosystem‐based management is a priority, as seen implemented throughout United States by the Environmental Protection Agency (Lewis et al., 2020), the scavenging community should be considered during planning. We also encourage consideration of this concept applied to the future management of freshwater fish species worldwide.

## 8 CONSERVATION OF SCAVENGERS

We must understand carcass acquisition and competition between obligate scavengers, facultative scavengers, and decomposers to effectively conserve rare scavengers and maintain properly functioning ecosystems. However, some competitive interactions, particularly competition between vertebrates, invertebrates, and microbes, are not well described (Beasley et al., 2015). For example, the endangered American burying beetle (*Nicrophorus americanus* Olivier) is an obligate scavenger that requires larger vertebrate carcasses than are used by congeners for feeding and reproductive purposes, resulting in increased competition with vertebrate scavengers in addition to other invertebrates such as flies and ants (Szalanski et al., 2000). Although extensive research has been conducted on American burying beetle ecology (Bedick et al., 1999), there are still many questions concerning the decline of this species and how to best employ conservation strategies (Howard & Hall, 2019), particularly in the case of scavenging competition that can be impacted directly and indirectly through management practices (DeVault et al., 2011). Additionally, several studies (Burkepile et al., 2006; DeVault et al., 2004) have elucidated the complexities of interkingdom competition at carcasses, indicating that microbes can chemically deter vertebrate scavengers from carrion; however, more information is needed to fully understand the competitive interactions between microbes, vertebrates, and invertebrates, and the roles microbes play in food webs.

Supplementary feeding stations (SFS) have been implemented globally and contributed to conservation strategies for many scavenging species. Vultures have benefitted from SFS and studies have documented increasing population numbers worldwide at these sites (Clements et al., 2013), likely due to the predictable nature of food resources. However, vulture species are also reported to have large home ranges extending well beyond the locations of SFS (Monsarrat et al., 2013). The predictable and prolonged availability of resources at SFS also represents a deviation from the more ephemeral and variable availability of resources that occurs naturally. Thus, although proximate increases in vulture numbers at SFS may be a desirable conservation outcome, the extent to which the structure and function of feeding guilds may be altered due to a shift in competition at SFS and the predictability of resources is an area where further study is needed (Cortés‐Avizanda et al., 2016). Specifically, we suggest focusing on reproductive success and diversity of the scavenging guild for populations sustained with SFS, and predation pressure for small‐ and medium‐sized prey species living in the same areas of feeding stations. Additionally, Cortés‐Avizanda et al. (2010) found that local characteristics and the differences in carcass size supplied at SFS can influence how scavenging species, primarily those of conservation concern, use the feeding stations. Providing small quantities of food could be advantageous for ecological relationships within the scavenger guild. The reoccurring and abundant resource availability could have cascading effects on the evolution of scavenger populations, and should be monitored long term and considered in conservation and management plans.

There are many apex predators that are facultative scavengers currently listed as vulnerable, threatened, or endangered species, such as the snow leopard (*Panthera uncia*), African lion (*P. leo*), and red wolf (*Canis rufus*). Despite the extensive efforts made to protect endangered and threatened animals globally, little research has been conducted to understand how carrion provisioning can play a role in facultative scavenger conservation, particularly for mammalian predators. Most predators are facultative scavengers, consuming carrion if and when it is available (DeVault et al., 2003; Wilson & Wolkovich, 2011), which can vary based on seasonality, diet, and habitat. Although some research has focused on carcass provisioning for scavenger conservation (Stiegler et al., 2020) and the benefits derived from providing supplemental carcasses (Benbow et al., 2018), there is little focus on mammalian predator species and how scavenger management could improve conservation strategies. Additionally, competition between other predators and carrion resource partitioning has been examined to further elucidate the importance of scavenging in food webs, as well as the trophic cascade that could occur with the removal of those predators (Wilmers et al., 2003). Examinations of inter‐specific interactions between apex predator populations provided insight into the importance of a diversity of carcass sizes for coexistence of large carnivores, especially in small protected preserve areas. However, given the paucity of data on the importance of carrion to many apex predators, studies are critically needed to better quantify scavenging dynamics and carrion acquisition in these species (Amorós et al., 2020) for consideration in conservation planning strategies for apex predators (Figure 2f).

## AUTHOR CONTRIBUTIONS

**Jessica Patterson:** Conceptualization (equal); investigation (equal); methodology (equal); writing – original draft (lead). **Travis DeVault:** Conceptualization (equal); writing – review and editing (equal). **James Beasley:** Conceptualization (equal); writing – review and editing (equal).

## CONFLICT OF INTEREST

The authors declare that they have no known competing financial interests or personal relationships that could have appeared to influence the work reported in this manuscript.

## Data Availability

Data availability is not applicable to this article as no new data were created or analyzed in this study.
